# Gastrocnemius Neuromuscular Activation During Standing Explosive Acceleration

**DOI:** 10.3390/life14111378

**Published:** 2024-10-26

**Authors:** Ana Ferri-Caruana, Carlos Sendra-Pérez, Jose Ignacio Priego-Quesada

**Affiliations:** 1 Prevention and Health in Exercise and Sport (PHES) Research Group, Department of Physical Education and Sports, University of Valencia, 46010 Valencia, Spain; ana.maria.ferri@uv.es; 2 Research Group in Sports Biomechanics (GIBD), Department of Physical Education and Sports, University of Valencia, 46010 Valencia, Spain; j.ignacio.priego@uv.es; 3 Department of Education and Specific Didactics, Jaume I University, 12006 Castellon, Spain; 4 Biophysics and Medical Physics Group (GIFIME), Department of Physiology, University of Valencia, 46010 Valencia, Spain

**Keywords:** calf, running, injury, muscle activation, EMG

## Abstract

The gastrocnemius muscle plays a crucial role in transmitting and generating energy during standing explosive accelerations, and as a consequence, is a muscle with high injury prevalence, especially the medial gastrocnemius (MG). This study aimed to compare the neuromuscular activation of the lateral gastrocnemius (LG) and MG during one of the most common standing explosive accelerations performed in team sports—the false start that occurs in jumps where the leg steps back before moving forward. Forty-two physically active participants (34 males: age = 24 ± 5 years, body mass = 73 ± 10.4 kg; and 8 females: age = 26 ± 5 years, body mass = 57.1 ± 6.8 kg) underwent electromyography analysis of the MG and LG in the four first foot contacts of standing explosive acceleration. The results showed that the third contact differed significantly from others (LG vs. MG: 76.48 ± 3.10 vs. 66.91 ± 2.25, *p* = 0.01, ES = 0.5), with the LG exhibiting earlier activation and higher peak sEMG activity compared to the MG (LG vs. MG: 0.12 ± 0.01 vs. 0.13 ± 0.01, *p* = 0.02, ES = 0.4). Additionally, the MG displayed longer duration contractions in all the foot contacts except the third foot contact. In conclusion, the MG showed an earlier activation timing and a longer duration of contraction than the LG in the first foot contact. Additionally, the third foot contact showed a different pattern of neuromuscular activation between the MG and LG compared to the rest of the foot contacts.

## 1. Introduction

The gastrocnemius acts as a transmitter of hip–ankle energies to produce mechanical effort as efficiently as possible during the explosive extensions of the leg [1]. This dynamic role involves the transmission and reception of energy, stabilizing knee and ankle joints, and propelling forces during activities such as sprint running [1,2]. Notably, in sprint running, the gastrocnemius plays a pivotal role in generating the essential horizontal force impulse within a brief time frame [3]. The gastrocnemius muscle is composed of two muscular bellies (lateral gastrocnemius (LG) and medial gastrocnemius (MG)), that are located in the same anatomical region [4,5]. Recent studies have revealed distinct characteristics between these two muscles, with the MG having a greater pennation angle and greater force production, while the LG shortens with higher velocity [4,6]. In addition, neural activation seems different between these muscles since they are activated independently by the nervous system depending on contraction type and/or joint configuration [7]. Different muscular activation patterns between MG and LG have been found during functional activities such as calf raise strength training with different foot positioning [8,9], during forward sways of the body in a human standing balance [10], and during standing external perturbations across different directions while walking and running [11,12,13].

The gastrocnemius muscle has very high injury prevalence in runners, with the MG having a higher incidence than the LG (58–65% vs. 8–38%) [14,15]. Nevertheless, these injuries seem to have multifactorial origins, including fatigability, cellular metabolism, and tendon stiffness [14]. These injuries often occur after sprinting over a short distance (5–10 m) where the aim is to produce the greatest horizontal acceleration from either a stationary or a moving position, which is a very common motor task in most team and split-court sports [14,16]. To better understand the specific activation patterns of the LG and MG through sEMG, studies can provide critical insights into how these muscles respond under different conditions, helping to identify potential weaknesses or imbalances that could contribute to injury [17]. For example, while soccer players sprint, their higher gluteal and trunk muscle activity during the airborne phases of sprinting have been associated with a lower risk of hamstring injuries [18].

Among the main types of standing explosive acceleration with the same start (i.e., with the feet in parallel) the following are included [19]: parallel starts, when the movement begins by advancing one foot; jump starts, when the movement begins with a backward jump with both feet; and false starts, that refer to jumps where the leg steps back before moving forward (also known as the “false step”) and are characterized by a quick step opposite to the desired direction of movement. However, although most of these common standing starts have been studied from a biomechanical point of view, the main focus has been to improve the performance [19,20]; limited research has focused on the neuromuscular activation of the gastrocnemius during these actions. Due to the relevance of calf injuries during short sprints and specifically, the higher injury incidence of the MG versus the LG, for this reason, a better understanding of their specific activation pattern during standing explosive acceleration is convenient. Moreover, focusing on describing the neuromuscular profile of gastrocnemius during the false start is interesting, as it has been shown to generate higher sprint performance than the other starting stances [21].

Therefore, the aim of this study was to evaluate the neuromuscular activation of the LG and MG, during the false start. We hypothesized that due to structural, functional, and neural differences in both bellies, MG would have a neuromuscular pattern different from LG.

## 2. Materials and Methods

### 2.1. Participants

This experimental study was performed with 42 physically active individuals (34 males: age = 24 ± 5 years, height = 179 ± 7 cm, body mass = 73.0 ± 10.4 kg and 8 females: age = 26 ± 5 years, height = 166 ± 6 cm, body mass = 57.1 ± 6.8 kg). A priori power analysis indicated that 40 participants would be sufficient to obtain a power of 0.80, with an alpha of 0.05, and a large effect size (Cohen’s f) of 0.40 (G*Power, version 3, University of Düsseldorf, Düsseldorf, Germany). The start and end of the recruitment period was in June 2022 and April 2023, respectively. Leg dominance was determined by asking the subjects which of their legs was their preferred leg to kick a ball with [22]. The inclusion criteria were (i) healthy participants between 18 and 40 years; (ii) physically active participants (at least 30 min/day, 3 times/week); and (iii) participants without any injuries in the last six months before the start of the study. Participants were not allowed to enroll if they had a body mass index greater than 30 kg/m^2^, a previous history of lower limb surgery, a neurological or autoimmune disease, any exercise contraindication, or refused to comply with the study protocol.

The study protocol was approved by the ethics committee of the University of Valencia (ref. 1973315) and performed in accordance with the latest revision of the Declaration of Helsinki. The participants were informed verbally and in writing about the procedures, possible risks, and benefits of the tests, and they provided written consent before the start of the study. 

### 2.2. Experimental Procedure

**Figure 1:**
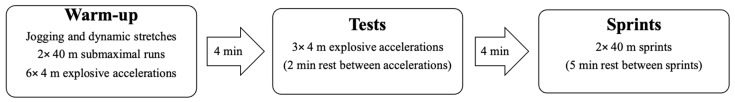
Study protocol timeline.

The participants attended the laboratory for a single-session testing protocol (Figure 1). The participants underwent a standardized warm-up consisting of an 8 min jog at a self-selected pace, 8 min of dynamic stretching including six repetitions for all the muscles involved (gluteus maximus, quadriceps, hamstrings, hip abductors and adductors, and gastrocnemius/soleus muscles), two submaximal runs of 40 m (approximately 80% of maximum perceived speed and rest of 3 min between runs) and finally, to become familiar with the tests, six repetitions of 4 m explosive accelerations (three with a standing dynamic start and three with a standing static start, with 1 min of rest between the repetitions). Once the warm-up ended, the participants rested for a 4 min period and performed three explosive accelerations over a 4 m distance with 2 min rest between the accelerations. All the participants received a brief pre-session explanation and a visual demonstration of the explosive acceleration.

Explosive accelerations consisted of participants standing straight with their feet shoulder width apart, their arms hanging naturally to the sides, and looking straight ahead. The participants received an auditive stimulus, and then performed the false start, which consisted of taking a step backwards to break inertia, and ran the 4 m distance as fast as they could. The signal was not given until the participant was completely in a neutral position with their body weight on both feet (not with a forward-inclined position since the tendency was to perform an anticipatory movement).

Finally, two repetitions of 40 m sprints were performed to normalize the sEMG signal. The participants started from a leaning standing start with one foot in front of the other one. At the sound of “ready” and “go”, they were expected to run as fast as they could until they reached the finishing mark. A 5 min active rest was set between the sprints. The measurements were performed on a standard cement surface with participants wearing their usual sports shoes.

### 2.3. Procedures

Two sEMG devices (MDurance Solutions S.L.; Granada, Spain) were used to collect muscle activity, using a bipolar electrode configuration for the acquisition of superficial muscle activity (1024 Hz) [23]. sEMG measurements were conducted by an experienced researcher following the SENIAM criteria [24]. First, the skin was shaved and cleaned with alcohol, then, surface electrodes were placed over the MG and LG of both legs with an inter-electrode distance of 10 mm (Kendall ^TM^ Medi-Trace; Coividien, Barcelona, Spain). The reference electrode was placed at the lateral malleolus of the fibula. Two synchronized two-channel handheld devices coupled with a Shimmer branch inertial sensor (Realtime Technologies Ltd; Dublin, Ireland) with 16-bit analog-to-digital (A/D) conversion were employed. Once the electrodes were placed at the muscle bellies and the electromyograph devices were allocated in the inner part of the tibia, a compressive tubular bandage was placed over the lower leg and the ankle to prevent electrode and sensor displacement.

### 2.4. Data Analysis

The participants performed three standing explosive accelerations. To determine which of the three starts was going to be further analyzed, the fastest standing explosive acceleration trial of each participant was selected. Video analysis with Kinovea (version 0.9.4) was used to measure the time required to reach a 4 m distance.

**Figure 2:**
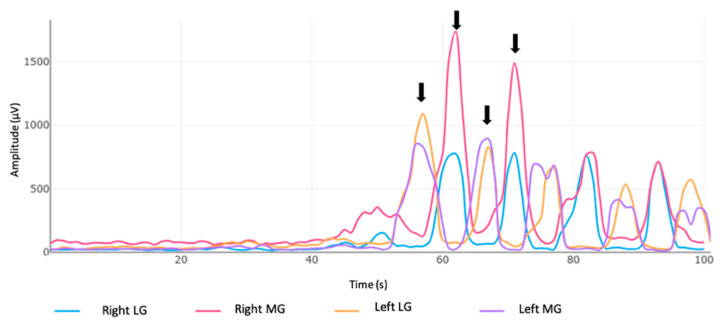
Electromyographic signal of the lateral and medial gastrocnemius muscles from a participant during the false start acceleration at each of the foot contacts. LG: lateral gastrocnemius; MG: medial gastrocnemius; s: seconds; μV: microvolts; black arrows indicate each of the first four foot contacts analyzed in the study.

sEMG signals from the explosive acceleration and the 40 m sprints were used for further analysis. The first four foot contacts (two with the right leg and two with the left leg) were analyzed, with the first foot contact considered as the lagging leg used for impulse generation. Figure 2 shows the sEMG activity of the LG and MG during the false start test, and it was measured for the four foot contacts of the starting position, including the step back.

The mDurance software (https://mdurance.com, (accessed on 1 February 2023)) digitally filtered the raw signals automatically using a fourth-order “Butterworth” bandpass filter between 20 and 450 Hz. A high-pass cut-off frequency of 20 Hz was employed to reduce any “artifacts” that might have occurred throughout the movement and that had a negligible impact on the total power recorded by the sEMG [25].

The sEMG data were normalized using the maximum activation (peak sEMG) of each muscle during the sprint of 40 m [26,27]. The mean of the two maximum values (throughout the sprint) was used for normalization. Therefore, all the data were presented as the percentage (%) of the maximum activation obtained during the sprint.

The variables obtained were the peak of sEMG activity, the start and end of muscle contraction, and the time to peak contraction. The difference between the start of the muscle contraction of the MG and the LG was used to calculate the timing of activation. The activation thresholds used to obtain these variables were (i) 2 time windows of 50 ms each (100 ms) and (ii) an amplitude of 18 % muscular activity. In this regard, the “time window” stablished the minimum period to ensure that a muscle has relaxed, and the “amplitude” set the intensity of muscle activation to establish the start and end of a contraction [28]. Because of the particularity of the contractions, the optimal threshold value used for the amplitude was 18% instead of 5%, as commonly used [29]. Additionally, the duration of contraction in each foot contact during the 4 m was calculated by subtracting the initial registered time from the final registered time for each foot contact.

### 2.5. Statistical Analysis

Statistical analyses were performed using RStudio (version 2022.02.03) with primary package “ggstatsplot”. The normality of data distribution was tested using the Shapiro–Wilk test and showed a non-normality distribution (*p* < 0.05). Wilcoxon tests were applied to assess the differences between both gastrocnemius in peak sEMG activity, and the time to peak sEMG activation at each foot contact. In addition, the timing of the four contacts and the duration of contraction in each foot contact were compared by applying a Kruskal–Wallis one-way using the timing of activation, and another Kruskal–Wallis one-way with the duration of the muscular contraction. A pairwise test was then performed using Durbin–Conover with a Bonferroni adjustment. Finally, to assess the magnitude of the significant pair differences, effect sizes (ES) (d of Cohen with Hedge correction) were computed using the package “effectsize” and classified as small (ES 0.2–0.5), moderate (ES 0.5–0.8), or large (ES > 0.8) [30].

## 3. Results

### 3.1. Differences in Peak sEMG and Time to Peak Activation Between Medial and Lateral Gastrocnemius

Similar MG and LG peak sEMG and time to peak sEMG values were found for the first, second, and fourth foot contacts. However, in the third foot contact, the LG showed higher peak sEMG (LG vs. MG, 79% and 67%, respectively, *p* = 0.01 and ES = 0.5) (Table 1) and an earlier time to peak activation than the MG.

### 3.2. Activation Timing

**Figure 3:**
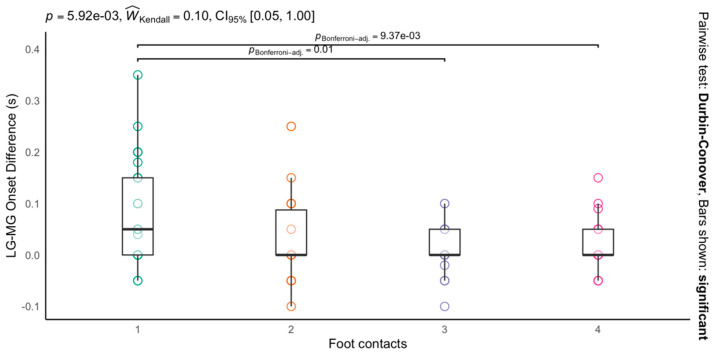
Differences in time of activation between medial and lateral gastrocnemius at each foot contact during a false start. Positive values indicate a slower activation onset for the lateral gastrocnemius (LG). Boxes represent interquartile ranges (IQR); whiskers extend to 1.5 times the IQR.

The timing of activation between the MG and LG showed differences in the first foot contact of the false start compared to the rest of the foot contacts (*p* < 0.001) (Figure 3). In this regard, the first foot contact showed a greater difference in activation timing (between the MG and the LG), than the third (first vs. third foot contacts, 0.08 ± 0.01 s vs. 0.01 ± 0.01 s; *p* < 0.01 and ES = 1.0) and fourth foot contacts (first vs. fourth foot contacts, 0.08 ±0.01 s vs. 0.01 ± 0.01 s; *p* < 0.01 and ES = 1.0). Specifically, the MG activated an average of 0.09 s earlier than the LG.

### 3.3. Duration of Muscular Contraction at Each Foot Contact in Both Gastrocnemius

**Figure 4:**
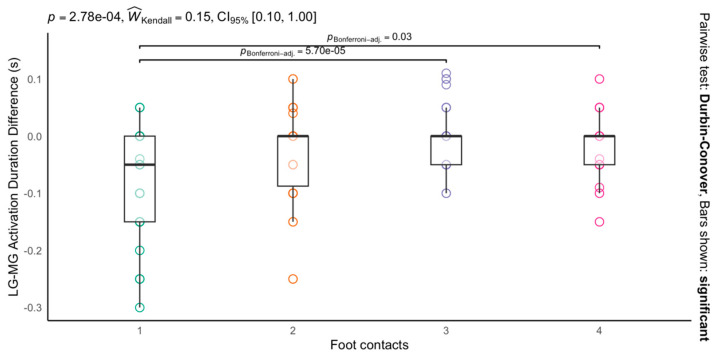
Differences in duration of muscular contraction in seconds between medial and lateral gastrocnemius at each foot contact during a false start. Negative values indicate a greater duration of the medial gastrocnemius (MG). Boxes represent interquartile ranges (IQR); whiskers extend to 1.5 times the IQR.

In terms of the duration of the muscular contraction of LG and MG at each foot contact, the results showed differences between the foot contacts (*p* < 0.001). The first foot contact showed a shorter duration of muscular contraction than the third (first vs. third foot contacts, −0.08 ± 0.01 s vs. −0.002 ± 0.01 s; *p* < 0.05 and ES = 1.1) and fourth foot contacts (first- vs. fourth-foot contacts, −0.08 ± 0.01 s vs. −0.02 ± 0.01 s; *p* < 0.001 and ES = 0.9) (Figure 4).

## 4. Discussion

The aim of this study was to evaluate the neuromuscular activation of the LG and MG during false-start explosive acceleration. The main finding was that MG and LG showed different neuromuscular activation at foot contacts one and three, supporting our hypothesis. Specifically, the LG showed a higher peak sEMG activation, and a longer duration and time to peak contraction than the MG muscle in the third foot contact. On the other hand, the MG showed an earlier activation timing, and a longer duration of contraction compared to the LG in the first foot contact (rear foot).

### 4.1. Differences in Peak sEMG and Time to Peak Activation Between Medial and Lateral Gastrocnemius

The vast majority of studies on MG and LG muscle activation reported values mainly from isometric and dynamic exercises, making it difficult to appreciate differences in functional explosive actions [10,31,32,33]. The overall consensus is that MG activation produces higher force than the LG due to its structural, functional, and neural difference [12]. Opposite to our study, very similar peak and time to peak muscular activation has been observed between both muscles at the first, second, and fourth foot contacts. A possible explanation could be the difference in muscle velocity contraction and the type of activity analyzed. Our results are from explosive contractions where the LG might be more relevant than MG considering their architectural differences, such as a lower pennation angle and longer fiber length, which would allow the LG muscle to generate force at a greater maximum shortening velocity. On this latter point, other studies have also noted muscle-specific, velocity-related differences in activation strategies in the three superficial quadriceps muscles, providing evidence of different muscle functions in relation to movement velocity within the same anatomical muscle group [34,35]. Therefore, differences in muscle function (isometric vs. dynamic and fast contractions) imposed by different mechanical constraints might have involved different neuronal circuits [36,37].

On the other hand, although the third and the second foot contact showed the highest mechanical loads, based on the higher muscle activation levels found, only the third one showed a difference in LG and MG neuromuscular activation (i.e., a longer time to peak activation in the MG). In this regard, differences in mechanics or central motor control centers that could affect motor unit recruitment patterns could explain this difference. During the first foot contacts of a standing explosive sprint the center of mass descends to break inertia for faster acceleration; this would imply higher knee and ankle flexion–extension moments in the second compared to the third foot contacts. The lower activation of MG at the third foot contact compared to the second one may have been due to the lower demand on the ankle function and the maintenance of muscular demands at the knee joint. According to Fiebert et al. [38] and Héroux et al. [10]. the LG and MG do not share the same optimal range of knee and ankle positions for force production, supporting the idea that LG, during knee flexion, might act more as a mediator, transferring energy between the knee and ankle, and the MG might have a greater response during plantar flexion at the ankle joint.

In addition, differences in neuromuscular activation in the LG and MG at the third foot contact compared to the second foot contact could be explained by motor control affected by limb dominance. In this regard, the motor function is different; the dominant leg is preferred for exercise, while the non-dominant leg works for stabilizing and supplementing the movements performed [39]. A recent study on the kinetics of bilateral lower limbs at different walking speeds showed that the impulse of the dominant lower limb increased significantly compared to the non-dominant leg during the propulsive phase of high-speed walking [40]. Considering that limb dominance is usually task-specific [41], this may affect the neuromuscular activation of the LG and MG found between the second and third foot contacts, because most of the participants were right-leg dominant. Further research is needed to understand the muscular event that was produced in the third foot contact since activation alone does not provide us with all of the information necessary to understand neuromuscular function.

### 4.2. Activation Timing

Muscle activation is a complex process that occurs in a short time frame during explosive acceleration. Differences in activation timing between both activation bellies should be considered as this action may be repeated several times during training [6]. In our study, the MG showed an earlier activation time, and a longer duration of contraction compared to the LG in the first foot contact (rear foot). A possible explanation for these results is the specific characteristics of the step backwards, which is used to break inertia. This foot contact tends to be short and lateral to the center of mass, implying maximum dorsiflexion with the knee extended, and the foot tends to be pointed outwards [19]. The variation in the orientation of the foot has effects on the involvement of the gastrocnemius bellies. An inward tip orientation increases LG activation, while an outward orientation increases MG involvement [42]. Regarding the feet position adopted during the heel raise task (i.e., neutral vs. internally rotated), it was found that it affected only the LG neural drive (i.e., delayed recruitment time) with no change for the MG [33]. Furthermore, considering that the MG shows more resistance to lengthening and has a stiffer subtendon compared to the LG, the central nervous system may choose to activate the MG earlier to enhance the catapult-like mechanism of the stiffer muscle–tendon unit and larger muscle to contribute the necessary inversion to allow a more efficient push-off [43,44]. The differences in timing and duration of activation during the first foot contact suggest the importance of the MG in the first push-off acceleration during standing explosive starts.

Regarding the duration of contraction, the MG showed a longer duration of contraction than the LG in all the foot contacts except for the third one [45]. As previously mentioned, the third foot contact showed a higher neuromuscular implication of LG, in terms of peak sEMG and time to peak sEMG, compared to the MG. So, this interplay of both contraction intensity and velocity needed to complete the required motor task would explain this difference.

### 4.3. Experimental Recommendations and Future Research

There are some experimental considerations and limitations. First, our results were limited to the standing explosive acceleration of participants with different types of foot strikes. Second, it is essential to note that the inclusion of eight females might potentially influence the results because the activation of the MG was observed to be higher in females compared to males [46]. We considered that our female sample was not enough to perform an analysis of the sex effect, which could be considered a limitation of our study. In addition, the inclusion of physically active participants might affect the activation timing or duration of muscular contractions. Future research is necessary to investigate acceleration or change in directions in different sports to assess muscle activation patterns. It is also necessary to investigate the activation in both gastrocnemius in various types of standing explosive acceleration and in more muscle groups (i.e., soleus) of the lower limb for a better understanding of activation timing during different actions.

## 5. Conclusions

The MG and LG showed different neuromuscular activation during the first and third foot contacts of the false start, supporting evidence that these muscles are not controlled primarily shared by neural drive. Specific attention should be paid to the earlier activation time obtained in the MG compared to the LG in the first foot contact, considering the higher muscle injuries presented in the MG compared to the LG in explosive accelerations.

## Figures and Tables

**Table 1:** Differences in peak sEMG and time to peak sEMG between medial and lateral gastrocnemius at each foot contact during a false start.

	Foot Contact	Lateral Gastrocnemius	Medial Gastrocnemius	*p*-Value (ES)
Peak sEMG (%)	First	64.69 ± 2.44	65.01 ± 2.65	0.72 (0.1)
	Second	76.34 ± 3.27	75.50 ± 3.26	0.91 (0.0)
	Third	76.48 ± 3.10	66.91 ± 2.25	0.01 (0.5)
	Fourth	68.86 ± 2.54	66.79 ± 2.56	0.62 (0.1)
Time to peak sEMG (s)	First	0.16 ± 0.01	0.20 ± 0.02	0.06 (0.4)
	Second	0.13 ± 0.01	0.16 ± 0.02	0.10 (0.4)
	Third	0.12 ± 0.01	0.13 ± 0.01	0.02 (0.5)
	Fourth	0.11 ± 0.01	0.13 ± 0.01	0.14 (0.4)

Note: ES = Effect sizes.

## Data Availability

The dataset generated and analyzed during this current study is available from the corresponding author upon reasonable request.
